# ACSL1 affects Triglyceride Levels through the PPARγ Pathway

**DOI:** 10.7150/ijms.42248

**Published:** 2020-02-24

**Authors:** Tingting Li, Xiangdong Li, Heyu Meng, Lili Chen, Fanbo Meng

**Affiliations:** Department of Cardiology China-Japan Union Hospital of Jilin University, Changchun, China 130033.

**Keywords:** ACSL1, Triglyceride, Lipid Metabolism, Myocardium Infarction, PPARr

## Abstract

In clinical cohort studies, high expression of long-chain acyl-coenzyme A synthetases 1 (ACSL1 gene) in peripheral white blood cells of patients with acute myocardial infarction (AMI) has been utilized as molecular markers of myocardial infarction diagnosis. The plasma triglyceride level of AMI patients is significantly higher than that of healthy individuals. We hypothesized that the high expression of ACSL1 increases the level of triglyceride, which is one of the pathogenesis of AMI promoted by ACSL1. In this report, cell culture based methods were adopted to test the hypothesis and further investigate the effect and mechanism of ACSL1 on lipid metabolism. In this study, liver cells of healthy individuals were cultured, the overexpression and the knockdown vectors of ACSL1 were constructed and transfected into liver cells. The transfection was verified at the mRNA and protein level. Intracellular triglyceride content was quantitatively analyzed using ELISA. Changes of genes related to lipid metabolism were subsequently measured through PCR array. Overexpression of ACSL1 led to higher gene expression and protein levels compared to control and the triglyceride content was significantly increased in overexpressing cells. The expression level of fatty acid oxidation pathway PPARγ was significantly down-regulated compared with the control group, as were genes associated with fatty acid synthesis pathways: SREBP1, ACC, FAS, and SCD1. ACSL1 knockdown decreased the content of triglyceride whereas PPARγ was up-regulated and SREBP1, ACC, FAS, and SCD1 were down-regulated compared with the control group. In summary, high expression of ACSL1 reduced fatty acid β-oxidation through the PPARγ pathway, thereby increasing triglyceride levels.

## Introduction

Acute myocardial infarction (AMI) refers to myocardial necrosis caused by acute, persistent ischemia and hypoxia due to coronary atherosclerosis. AMI is a complex disease caused by the interaction of multiple factors, such as genetics, immunity and environment 1. Family history of coronary heart disease increases the risk of AMI. The occurrence of AMI shows obvious familial aggregation, and molecular genetic data shows that a large number of genes are associated with the occurrence of myocardial infarction 2. Dyslipidemia is a known risk factor for coronary heart disease and myocardial infarction 3. The levels of total cholesterol, plasma triglyceride and plasma low-density lipoprotein (LDL) of AMI patients are significantly higher than those of healthy individuals. The results of our previous gene chip study suggested that the expression of ACSL1 in peripheral white blood cells (PBL) of AMI patients and healthy controls groups significantly increased (LogFC=2.590, P=0.04) 4. The long-chain acyl-coenzyme A synthetases (ACSL) family played a key role in regulating fatty acids which entered the synthetic or oxidative pathways 5. ACSL catalyze fatty acids to generate energy or produce phospholipids, cholesterol esters, and triglycerides 6. The distribution of intracellular fatty acids in storage pathways and oxidation might also involve different isozyme forms of acyl-coenzyme A synthase (ACSL) 7. The long-chain acyl-coenzyme A synthetases 1 (ACSL1) is one of the five clonal isomers of ACSL 8.

ACSL1 exists in cells of the liver, heart and fat 9, and is the most abundant acyl-coenzyme A synthetases subtype in adipose tissue, liver and heart, and has a wide range of fatty acid specificity 10,11. By using allotype specific antibodies, ACSL1 has been located in the endoplasmic reticulum, mitochondrial related membrane and cytoplasmic matrix, but not in other components of the mitochondria 6. ACSL1 is considered to play an important role in activating fatty acid synthesis of triglyceride 12. Overexpression of ACSL1 in mouse hearts increased the accumulation of triglyceride in cardiomyocytes by 12-fold 13. Other studies showed that ACSL activity was strongly correlated with changes in liver cholesterol and free cholesterol in plasma 14.

Previous cohort studies indicated that the expression of ACSL1 in peripheral white blood cells of AMI patients increased compared with that of the healthy control group. The high expression of ACSL1 was an independent risk factor for AMI. The triglycerides content in peripheral white blood cells of patients with myocardial infarction was shown to increase 4. Therefore, the high expression of ACSL1 gene increased the level of triglyceride, which has been hypothesized to be one of the pathogenesis of AMI promoted by ACSL1. Therefore, cultured healthy human liver cells and cell culture-based assays were adopted to test the hypothesis in this study.

## Materials and methods

### Experimental Materials

Human liver cells and 1640 medium were purchased from Zhongqiao Xinzhou Biotechnology Co. Ltd (Shanghai, China). Australian fetal bovine serum and dual antibody were purchased from Invitrogen, Carlsbad, CA, USA and transfection reagents were purchased from Promega (Beijing) Biotech Co., Ltd. ACSL1 monoclonal antibody (1: 1000, ab177958) was purchased from Abcam, Cambridge, MA, USA.

## Experimental Methods

### Vector Construction

Vectors which over express or under express ACSL1 were synthesized by GenePharma Co. Ltd., (Shanghai, China) and were designed according to the ACSL1 mRNA sequence of human cells (NM_001995) published by the NCBI website. The empty vectors encoding for green fluorescent protein (GFP) were used as the negative control.

### Cell Culture

Human hepatocytes were cultured at a constant temperature of 37 °C in a 5%CO_2_ incubator. The 1640 medium was used (containing 90% medium, 10% Australian fetal bovine serum and 100U/ml dual-antibody). When the confluency of cells reached 70% to 80%, the cells were harvested with trypsin and cultured for another 24 hours.

### Cell Transfection

Cells were transfected when the confluency reached over 70%. 3μg pBI-CMV3-ACSL1, pBI-CMV3, 3 μg pGPU6/GFP/Neo-ACSL1, or pGPU6/GFP/Neo-shNC vectors were added to 150μl serum-free RPMI 1640 media. 7.5 μl FuGENE®HD Transfection Reagent was added to the solution before being incubated for 15 minutes. The mixture was evenly dripped clockwise into a six-well plate and placed in the incubator. After 24 hours of culture, the cell state and distribution of green fluorescent protein could be observed under a fluorescence microscope.

### Western blotting

Protein lysates were prepared 48 hours after transfection, and the protein concentration was measured using the BCA protein detection kit. 30 μg of protein samples were denatured at 95 °C for 5 minutes. After SDS-PAGE electrophoresis the proteins were transferred onto a PVDF membrane at 200 mA for 60 min. The membrane was blocked at room temperature with a blocking buffer (5% skimmed milk powder) for 1 hour. Subsequently, the membranes were incubated with the primary antibody overnight at 4 °C. After washing, the membrane was then incubated with the secondary antibody at room temperature for 1 hour and the signal was developed using a chemiluminescent imaging system.

### The mRNA expression of ACSL1 genes and lipid metabolism-related genes were detected using PCR array technology

24 hours after the cells were transfected, total RNA was extracted using the innuPREP RNA Mini Kit (Sangon Biotech Co., Ltd, Shanghai, China). The RNA quality of the collected samples was tested by agarose gel electrophoresis. The concentration and purity of RNA were tested using a nanodrop spectrophotometer (NanoDrop 2000; Thermo Fisher Scientifc Inc., Wilmington, DE, USA). cDNA was obtained using reverse transcription kit (Toyobo Co., Ltd., Osaka, Japan). Quantitative real-time PCR was conducted using SYBR^®^ Premix Ex Taq. The primer sequences are shown in Table 1. Quantitative PCR was performed in triplicates on ACSL1 peroxisome proliferators-activated receptorsγ (PPARγ), and the following fatty acid oxidation-associated genes: sterol regulatory element-binding protein (SREBP1), acetyl-coenzyme A carboxylase (ACC), fatty acid synthase (FAS), stearyl carboxylase A dehydrogenase (SCD1) with GAPDH as the reference. The thermal cycling condition was at 50 °C for 2 minutes, at 95˚C for 10 minutes; at 95 °C for 10 seconds, at 60˚C for 30 seconds, at 72 °C for 15 seconds with 35 cycles, at 55 °C for 15 seconds, at 95 °C for 15 seconds. The Ct value of RT-qPCR of each sample was the average standard deviation of the triplicate, and the results were quantified by using 2-∆∆C.

### Triglyceride content detection

The tissue cell triglyceride enzyme assay kit (Applygen Technologies Inc.) was used to measure triglyceride content. After cell lysis, 20 μl supernatant was mixed with the working solution. The elisa plates were shaken gently and incubated at 37 °C for 10 minutes. The OD value of the samples was tested (Infinite 200 PRO, multifunctional enzyme marking instrument, the Swiss Tecan).

### Statistical Analysis

Data were analyzed using SPSS 22.0 (SPSS Incorporated, Chicago, IL, USA). Statistical analysis of the results and mapping were performed through GraphPad Prism 7.0. The results were expressed as mean ± standard deviation (SD). Independent sample *t*-test was used to compare differences between groups, using *P* <0.05 as the level of statistical significance.

## Experimental Results

### ACSL1 overexpression and knockdown vectors were transfected into human hepatocytes

**Figure 1:**
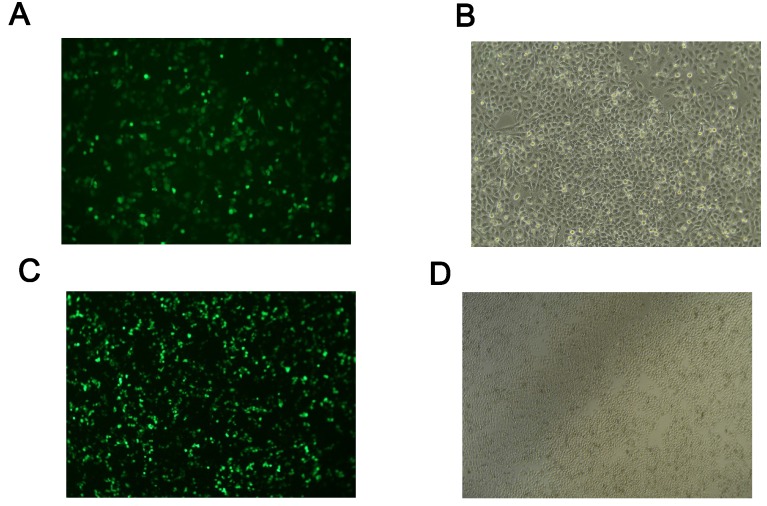
Transfection of hepatocytes with overexpression and knockdown vectors: (**A, B**) the fluorescence results of ACSL1 overexpressed vector cells under fluorescence microscope. (**C, D**) The fluorescence results of ACSL1 downregulated cells under fluorescence microscope.

After transfection of human hepatocytes with ACSL1 RNAi and overexpression vector for 24h, cell morphology, and fluorescence expression levels were observed under a fluorescence microscope. Green fluorescence could be observed in cells transfected with the pBI-CMV3-ACSL1 overexpression vector and the empty vector of pBI-CMV3, as well as the pGPU6/GFP/Neo-ACSL1 knockdown vector and the pGPU6/GFP/Neo-shNC negative control vector. GFP was evenly distributed in the Petri dish (Fig. 1).

### Western blot analysis

**Figure 2:**
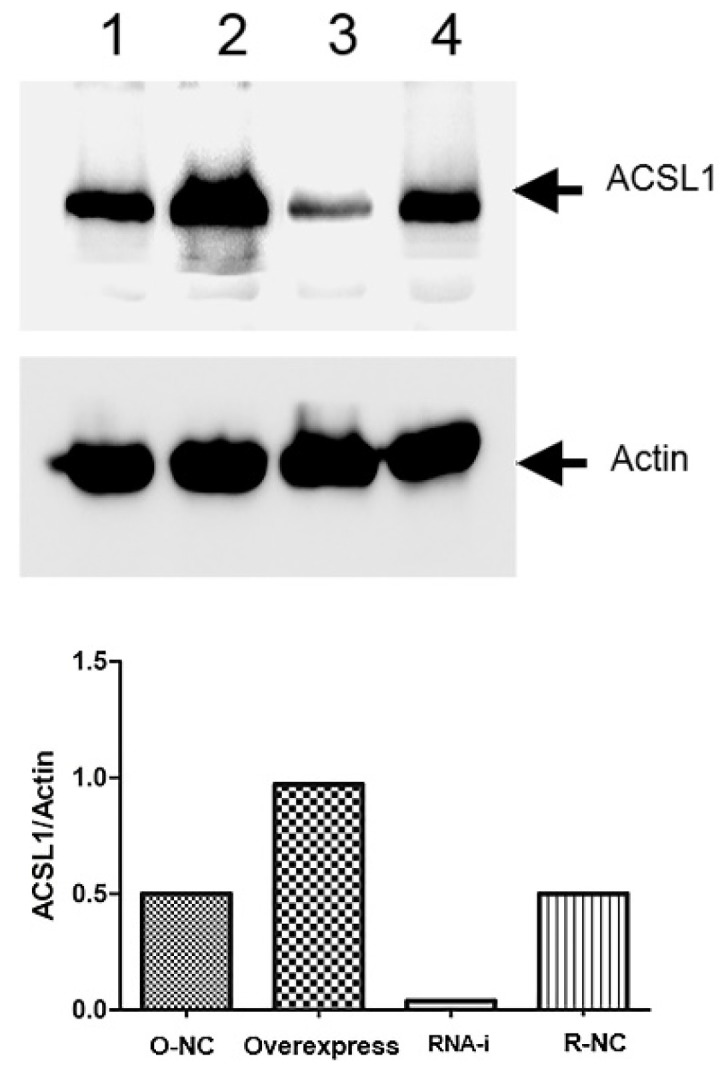
Comparison of expression level of ACSL1 gene at the protein level. Sample 1 is the overexpression control group, sample 2 is the overexpression group, sample 3 is the knockdown group, and sample 4 is the knockdown control group.

Western blot results showed that the expression of ACSL1 protein in the overexpression group was significantly higher than in the negative group. The expression of ACSL1 protein in the knockdown group was lower than in the negative group (Fig. 2).

### Analysis of ACSL1 gene overexpression results through RT-qPCR

**Figure 3:**
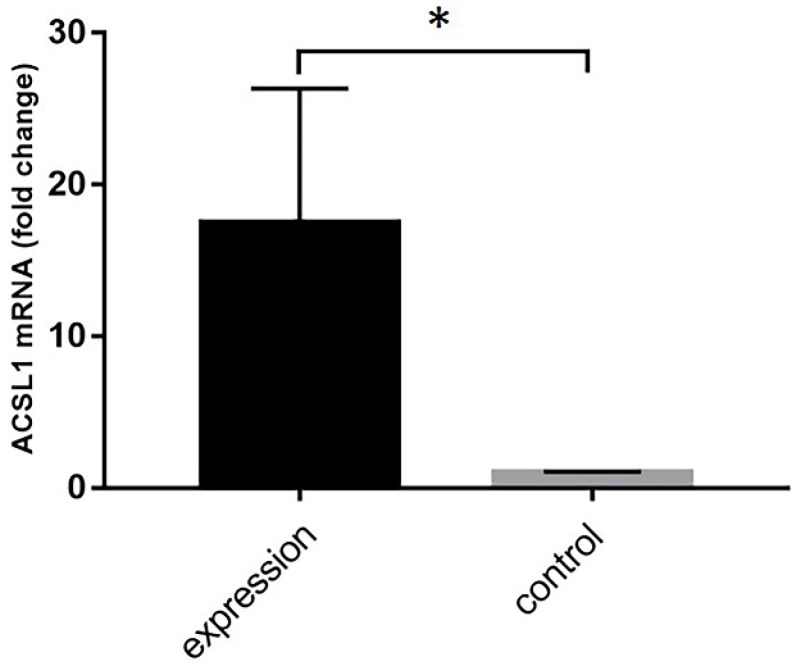
mRNA expression results of ACSL1 overexpression. *P <0.05.

The expression of ACSL1 gene overexpression vector was detected through qPCR. The overexpression of ACSL1 in liver cells was significantly higher than that of the control group (17.49 ± 5.10 vs 1.00± 0.05, *P*=0.032) (Fig. 3).

### Triglyceride content after ACSL1 gene overexpression

**Figure 4:**
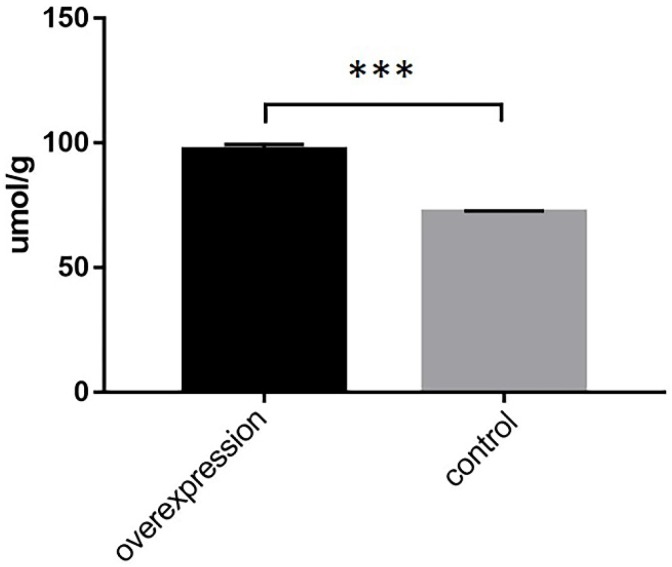
Determination of intracellular triglyceride content after transfection with an overexpressed vector. ***P < 0.001.

Triglyceride content was significantly increased in ACSL1-overexpressing cells (97.27 ± 2.22 vs 72.35 ± 0.44, *P*<0.001) (Fig. 4).

### Analysis of lipid metabolism related genes after ACSL1-overexpression

**Figure 5:**
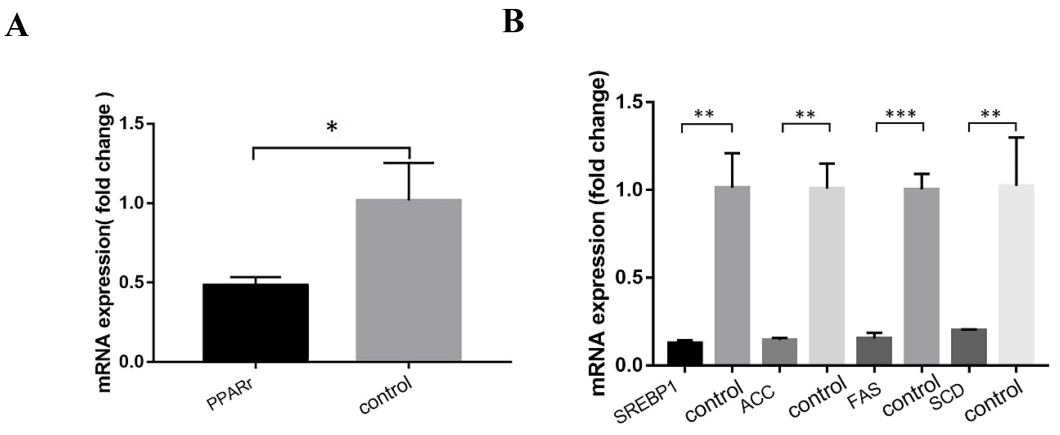
** (A)** Expression of genes related to fatty acid oxidation pathway after ACSL1 overexpression. **(B)** Expression of genes related to fatty acid synthesis pathway after overexpression of ACSL1. *P < 0.05, **P < 0.01, ***P < 0.001.

Relative expressions of lipid metabolism pathway genes in ACSL1-overexpression cells, compared to the control group were as follows: Fatty acid oxidation pathway, PPARr 0.48 ± 0.05 vs 1.02 ± 0.24, (*P*=0.019). The relative expression levels of fatty acid synthesis pathway related genes were as follows: SREBP1 0.13 ± 0.02 vs 1.01 ± 0.20, (*P*=0.001), SCD 0.20 ± 0.01 vs 1.02 ± 0.28, (*P*=0.007), ACC 0.14 ± 0.12 vs 1.00 ± 0.14, *(P*=0.001), FAS 0.15 ± 0.03 vs 1.00 ± 0.09, (*P*<0.001) (Figs. 5 A & B).

### Analysis of RT-qPCR results after downregulation of ACSL1

**Figure 6:**
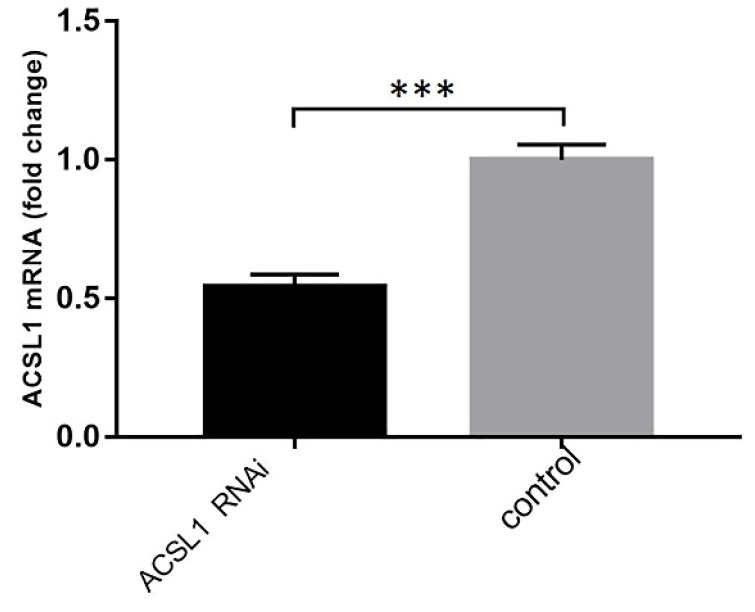
mRNA expression results of ACSL1 downregulation. ***P < 0.001.

The expression of ACSL1 downregulation vector was detected through qPCR. The expression level of ACSL1 gene mRNA in liver cells was significantly lower than in the control group. (0.54 ± 0.03 vs 1.00 ± 0.03, *P*<0.001) (Fig. 6).

### Triglyceride content after ACSL1 downregulation

**Figure 7:**
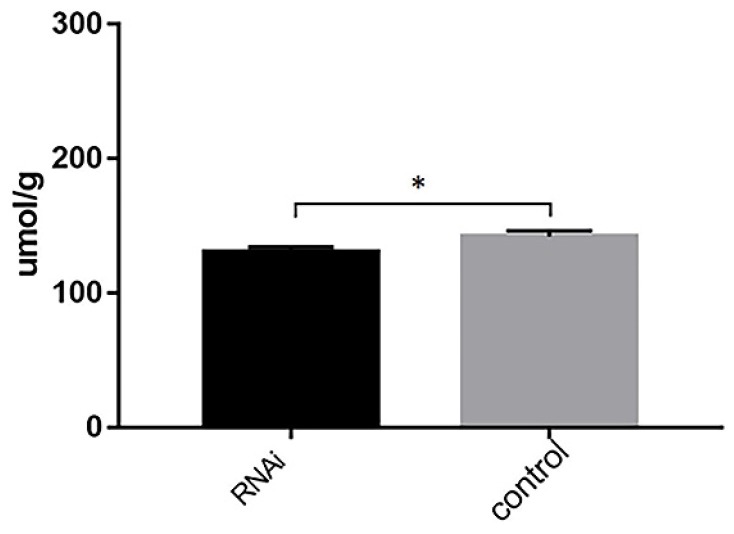
Triglyceride content in hepatocytes after hepatocytes were transfected with knockdown vectors. *P < 0.05.

Triglyceride content decreased when ACSL1 expression was downregulated (130.65 ± 3.64 vs 142.07 ± 4.23, *P*=0.024) (Fig. 7).

### Analysis of lipid metabolism-related genes after ACSL1 downregulation

**Figure 8:**
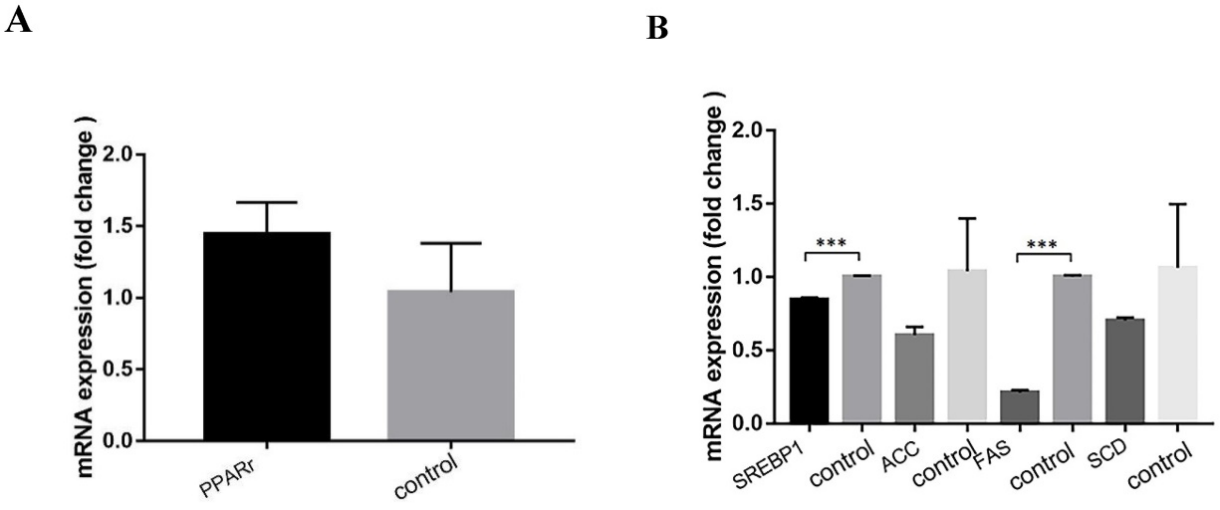
** (A)** Expression of genes related to fatty acid oxidation pathway after ACSL1 knockdown. **(B)** Expression of genes related to fatty acid synthesis pathway after ACSL1 knockdown. ***P < 0.001.

Compared with the control group, the relative expression levels of genes related to lipid metabolism pathway were as follows after ACSL1 downregulation: Fatty acid oxidation pathway PPARr 1.44 ± 0.22 vs 1.04±0.34(*P*=0.158). The relative expression levels of genes related to fatty acid synthesis pathway were as follows: SREBP1 0.85 ± 0.03 vs 1.00 ± 0.01 (*P*<0.001), FAS 0.21 ± 0.02 vs 1.00 ± 0.01 (*P*<0.001), SCD 0.70 ± 0.02 vs 1.06 ± 0.44 (*P*=0.226), ACC 0.60 ± 0.06 vs 1.04 ± 0.36 (*P*=0.107) (Figs. 8A& B).

## Discussion

Preliminary work of this research group indicated that the expression level of ACSL1 in peripheral white blood cells of AMI patients was significantly higher than that of healthy individuals, while the level of triglyceride in peripheral blood plasma of patients with heart infarction increased. In this study, healthy human hepatocytes were used, and cell culture-based assays were adopted to confirm that the triglyceride increased after ACSL1-overexpression, and decreased after ACSL1-downregulation.

Lipid accumulation in the liver was due to a combination of reduced fatty acid β-oxidation and increased fat synthesis 15. This study indicated that human hepatocellular carcinoma cells (HepG2) overexpressing ACSL1 had 40% higher triglyceride content than the control group after 24 hours of exposure to 1.0mM oleic acid ester. The cultivation of HepG2 ACSL1-overexpressing cells increased the uptake of fatty acids in the medium by 43% compared with the control group, and ^14^C oleate incorporation in the extracted lipids increased by 32%. However, where no significant change was observed, ^14^C oleate was transferred into CO_2_ or ASM (Acid soluble metabolites), which indicated overexpression of ACSL1 increased intracellular triglyceride levels by increasing intracellular fatty acid synthesis rather than by altering fatty acid oxidation 6.

Fatty acids, an important source of energy for mammals, are chemically inert and need to be activated by ACSL1 in cells outside of the mitochondria to form acyl-coenzyme A before they enter the metabolic pathway 16. Once activated to acyl-coenzyme A, fatty acids have multiple cellular fates, and the two main metabolic pathways are degradation by β-oxidation or incorporation into complex lipids 17. Because cells do not metabolize the energy required to oxidize fatty acids, acyl-coenzyme A is esterified into triglycerides. It has been reported that ACSL1 is related to the endoplasmic reticulum, plasma membrane, GLUT4 vesicles and cytoplasm rather than mitochondria, and thus, is more related to the synthesis pathway of fatty acid metabolism 18. This study, however, found ACSL1 to regulate triglyceride levels by affecting the oxidation pathway of fatty acids.

This study indicates that overexpression of ACSL1 in human liver cells results in decreased expression of the fatty acid oxidation-related gene PPARγ, thereby reducing the fatty acids β-oxidation and increasing triglyceride levels. PPARγ is upregulated after ACSL1 downregulation, thereby reducing triglyceride levels.

It was shown that Peroxisome proliferators which activate receptor-α (PPAR α) and PPARγ are associated with inducing ACSL1 expression in the heart and adipose tissue, and have an essential role in β-oxidation 19,20. In order to promote and maintain a mature adipose cell phenotype, the PPARγ ligand continues to activate fatty acid transporter protein (FATP) and acyl-coenzyme A synthetase (ACS), which results in increased delivery of fatty acids to adipocytes. This may be related to regulating feedback loops involving continued PPARγ activation of the FATP and ACS genes. This regulation of FATP and ACS expression by PPAR activators are found at the transcriptional level and can be replicated in vitro in cell culture systems 20.

PPARγ is a member of the peroxisome proliferation-activated receptor (PPAR) subfamily. Three types of PPAR subtypes are known: ppar-α, ppar-δ, and ppar-γ, with different distributions and specific functions 21. PPARγ is a marker gene that induces early adipocyte differentiation, and its coding is involved in lipid storage and metabolic protein synthesis during adipocyte differentiation. Several genes that have been shown to play a key role in fatty acid metabolism contain response elements of peroxisome proliferators in their upstream regulatory sequences 22. It has been proven that the destruction of liver PPARγ exacerbates hyperlipidemia by reducing the fatty acids β-oxidation and damaging the triglyceride clearance rate 23.

In this study, it was also found that pathway genes related to fatty acid synthesis, such as SREBP1, ACC, FAS and SCD1, are significantly down-regulated after overexpression of ACSL1. No consistent results are found after ACSL1 downregulation. Therefore, whether ACSL1 gene affects triglyceride metabolism through the fatty acid synthesis pathway is still uncertain. However, our study confirms that ACSL1 gene affects plasma triglyceride levels through PPARγ.

Sterol regulatory element-binding proteins (SREBPs) consist of three closely related transcription factors SREBP-1a, SREBP-1c and SREBP-2 24. A study on gene knockout and transgenic mice has shown that SREBP-1c preferentially regulates genes involved in fatty acid biosynthesis, while SREBP-1a and SREBP-2 mainly regulate genes in the cholesterol pathway 25. Therefore, SREBPs are involved in the synthesis of fatty acids and cholesterol. SREBP-1c is a major transcription regulator of lipid synthesis. It has been shown in cows that increasing the expression of SREBP-1c in liver correlates with lipid accumulation 26. In addition, the expression of SREBP-1c increased significantly in the case of liver triglyceride accumulation 27. ACC1, FAS, and SCD1 are key enzymes in lipid synthesis, and are also the target genes of SREBP-1c 28. ACC1 converts acetyl-CoA to malonyl CoA 29, which participates in the synthesis of fatty acids. FAS is the gene that determines the amount of *de novo* fatty acids produced in the tissue 30. SCD1 catalyzes the extension and desaturation of fatty acids 30,31.

SREBPs are conserved from fission yeast to humans, which regulate the expression of genes that control cell lipid homeostasis, depending on species-specific requirements. Therefore, these proteins and their target genes perform complex feedback regulation on SREBP activity at multiple levels 32. It was shown that overexpression of SREBP-1c can significantly up-regulate the expression of ACSL1 and increase the enzyme activity of ACSL1, thereby increasing the uptake and activation of liver fatty acids 33. In this study, the level of triglyceride increased significantly after the overexpression of ACSL1 compared with the control group. The low expression of SREBP1 and its target genes may be related to complex feedback regulation, but after ACSL1 downregulation, the results of fatty acid synthesis pathway-related genes (SREBP1, ACC, FAS, SCD1) and overexpression of ACSL1 are not consistent. The gene pathways involved in lipid metabolism are complex. Although this study has not confirmed whether ACSL1 gene influences triglyceride levels through the fatty acid synthesis pathway, we have demonstrated that ACSL1 gene influences triglyceride levels through the fatty acid β-oxidation pathway. Previous research has shown that ACSL1 overexpression can increase the synthesis of fatty acids in cells without significantly changing the oxidation of fatty acids 6, which is not completely consistent with our research results.

The liver is an important organ involved in lipid metabolism. Primary human hepatocytes are considered as the gold standard for investigating the molecular mechanisms involved in the development of nonalcoholic fatty liver disease (NAFLD) 34. It is known that cells from healthy human livers contain lower amounts of intracellular triglycerides than cells from hepatocellular carcinoma (HepG2) 35. Due to the availability, usability and donor variability of human liver cells, Hepatocyte cells are used as an alternative model for lipid metabolism in human liver in most studies of lipid metabolism 34. Liver cancer cells have shortcomings in the study of lipid metabolism, so it is more meaningful to adopt healthy human liver cells as the vector of ACSL1 gene and triglyceride metabolism and mechanism.

## Conclusion

The ACSL1 gene affects triglyceride levels.

The high expression of ACSL1 gene decreases fatty acid β-oxidation by PPARγ pathway, which increases the level of triglycerides.

Therefore, this research confirms that the high expression of ACSL1 gene, which increases the level of plasma triglyceride, is one of the mechanisms that promote the incidence of AMI.

## Figures and Tables

**Table 1:** Primer sequences

Gene names	Primer sequences (5'-3')
**ACSL1**	
F	CCATGAGCTGTTCCGGTATTT
R	CCGAAGCCCATAAGCGTGTT
**SREBP1**	
F	CGGAACCATCTTGGCAACAGT
R	CGCTTCTCAATGGCGTTGT
**SCD**	
F	TTCCTACCTGCAAGTTCTACACC
R	CCGAGCTTTGTAAGAGCGGT
**ACC**	
F	ATGTCTGGCTTGCACCTAGTA
R	CCCCAAAGCGAGTAACAAATTCT
**PPARγ**	
F	ACCAAAGTGCAATCAAAGTGGA
R	ATGAGGGAGTTGGAAGGCTCT
**FAS**	
F	AAGGACCTGTCTAGGTTTGATGC
R	TGGCTTCATAGGTGACTTCCA
**GAPDH**	
F	GGAGCGAGATCCCTCCAAAAT
R	GGCTGTTGTCATACTTCTCATGG

F, forward; R, reverse.

## Abbreviations

ACSL1: long-chain acyl-coenzyme A synthetases 1
ACC: acetyl-coenzyme A carboxylase
FAS: fatty acid synthase
PPARγ: peroxisome proliferators-activated receptors γ
SREBP1: sterol regulatory element binding protein
SCD1: stearyl carboxylase A dehydrogenase

## References

[1] Moreira DM, da Silva RL, Vieira JL, Fattah T, Lueneberg ME, Gottschall CA (2015). Role of vascular inflammation in coronary artery disease: potential of anti-inflammatory drugs in the prevention of atherothrombosis. Inflammation and anti-inflammatory drugs in coronary artery disease. American journal of cardiovascular drugs: drugs, devices, and other interventions.

[2] Zdravkovic S, Wienke A, Pedersen NL, de Faire U (2007). Genetic susceptibility of myocardial infarction. Twin research and human genetics: the official journal of the International Society for Twin Studies.

[3] Boateng S, Sanborn T (2013). Acute myocardial infarction. Disease-a-month: DM.

[4] Yang L, Yang Y, Si D, Shi K, Liu D, Meng H (2017). High expression of long chain acyl-coenzyme A synthetase 1 in peripheral blood may be a molecular marker for assessing the risk of acute myocardial infarction. Experimental and Therapeutic Medicine.

[5] Coleman RA, Lewin TM, Muoio DM (2000). Physiological and nutritional regulation of enzymes of triacylglycerol synthesis. Annual review of nutrition.

[6] Parkes HA, Preston E, Wilks D, Ballesteros M, Carpenter L, Wood L (2006). Overexpression of acyl-CoA synthetase-1 increases lipid deposition in hepatic (HepG2) cells and rodent liver in vivo. American Journal of Physiology-Endocrinology and Metabolism.

[7] Wang YL, Guo W, Zang Y, Yaney GC, Vallega G, Getty-Kaushik L (2004). Acyl coenzyme a synthetase regulation: putative role in long-chain acyl coenzyme a partitioning. Obesity research.

[8] Mashek DG, Li LO, Coleman RA (2006). Rat long-chain acyl-CoA synthetase mRNA, protein, and activity vary in tissue distribution and in response to diet. Journal of lipid research.

[9] Oikawa E, Iijima H, Suzuki T, Sasano H, Sato H, Kamataki A (1998). A novel acyl-CoA synthetase, ACS5, expressed in intestinal epithelial cells and proliferating preadipocytes. Journal of biochemistry.

[10] Suzuki H, Kawarabayasi Y, Kondo J, Abe T, Nishikawa K, Kimura S (1990). Structure and regulation of rat long-chain acyl-CoA synthetase. The Journal of Biological Chemistry.

[11] Lobo S, Wiczer BM, Bernlohr DA (2009). Functional analysis of long-chain acyl-CoA synthetase 1 in 3T3-L1 adipocytes. The Journal of Biological Chemistry.

[12] Li LO, Ellis JM, Paich HA, Wang S, Gong N, Altshuller G (2009). Liver-specific loss of long chain acyl-CoA synthetase-1 decreases triacylglycerol synthesis and beta-oxidation and alters phospholipid fatty acid composition. The Journal of Biological Chemistry.

[13] Chiu HC, Kovacs A, Ford DA, Hsu FF, Garcia R, Herrero P (2001). A novel mouse model of lipotoxic cardiomyopathy. The Journal of clinical investigation.

[14] Singh AB, Kan CF, Dong B, Liu J (2016). SREBP2 Activation Induces Hepatic Long-chain Acyl-CoA Synthetase 1 (ACSL1) Expression in Vivo and in Vitro through a Sterol Regulatory Element (SRE) Motif of the ACSL1 C-promoter. The Journal of Biological Chemistry.

[15] Zhu Y, Liu G, Du X, Shi Z, Jin M, Sha X (2019). Expression patterns of hepatic genes involved in lipid metabolism in cows with subclinical or clinical ketosis. J Dairy Sci.

[16] Mashek DG, Coleman RA (2006). Cellular fatty acid uptake: the contribution of metabolism. Current opinion in lipidology.

[17] Yan S, Yang XF, Liu HL, Fu N, Ouyang Y, Qing K (2015). Long-chain acyl-CoA synthetase in fatty acid metabolism involved in liver and other diseases: an update. World Journal of Gastroenterology.

[18] Lewin TM, Kim JH, Granger DA, Vance JE, Coleman RA (2001). Acyl-CoA synthetase isoforms 1, 4, and 5 are present in different subcellular membranes in rat liver and can be inhibited independently. The Journal of Biological Chemistry.

[19] Ellis JM, Li LO, Wu PC, Koves TR, Ilkayeva O, Stevens RD (2010). Adipose acyl-CoA synthetase-1 directs fatty acids toward beta-oxidation and is required for cold thermogenesis. Cell metabolism.

[20] Martin G, Schoonjans K, Lefebvre AM, Staels B, Auwerx J (1997). Coordinate regulation of the expression of the fatty acid transport protein and acyl-CoA synthetase genes by PPARalpha and PPARgamma activators. The Journal of Biological Chemistry.

[21] Leonardini A, Laviola L, Perrini S, Natalicchio A, Giorgino F (2009). Cross-Talk between PPARgamma and Insulin Signaling and Modulation of Insulin Sensitivity. PPAR research.

[22] Schoonjans K, Staels B, Auwerx J (1996). Role of the peroxisome proliferator-activated receptor (PPAR) in mediating the effects of fibrates and fatty acids on gene expression. Journal of lipid research.

[23] Gavrilova O, Haluzik M, Matsusue K, Cutson JJ, Johnson L, Dietz KR (2003). Liver peroxisome proliferator-activated receptor gamma contributes to hepatic steatosis, triglyceride clearance, and regulation of body fat mass. The Journal of Biological Chemistry.

[24] Eberle D, Hegarty B, Bossard P, Ferre P, Foufelle F (2004). SREBP transcription factors: master regulators of lipid homeostasis. Biochimie.

[25] Brewer M, Lange D, Baler R, Anzulovich A (2005). SREBP-1 as a transcriptional integrator of circadian and nutritional cues in the liver. Journal of biological rhythms.

[26] Prodanovic R, Koricanac G, Vujanac I, Djordjevic A, Pantelic M, Romic S (2016). Obesity-driven prepartal hepatic lipid accumulation in dairy cows is associated with increased CD36 and SREBP-1 expression. Research in veterinary science.

[27] Li X, Huang W, Gu J, Du X, Lei L, Yuan X (2015). SREBP-1c overactivates ROS-mediated hepatic NF-kappaB inflammatory pathway in dairy cows with fatty liver. Cellular signalling.

[28] Porstmann T, Griffiths B, Chung YL, Delpuech O, Griffiths JR, Downward J (2005). PKB/Akt induces transcription of enzymes involved in cholesterol and fatty acid biosynthesis via activation of SREBP. Oncogene.

[29] Wakil SJ, Stoops JK, Joshi VC (1983). Fatty acid synthesis and its regulation. Annual review of biochemistry.

[30] Postic C, Girard J (2008). Contribution of de novo fatty acid synthesis to hepatic steatosis and insulin resistance: lessons from genetically engineered mice. The Journal of clinical investigation.

[31] Man WC, Miyazaki M, Chu K, Ntambi J (2006). Colocalization of SCD1 and DGAT2: implying preference for endogenous monounsaturated fatty acids in triglyceride synthesis. Journal of lipid research.

[32] Shimano H, Sato R (2017). SREBP-regulated lipid metabolism: convergent physiology - divergent pathophysiology. Nature reviews Endocrinology.

[33] Li X, Li Y, Yang W, Xiao C, Fu S, Deng Q (2014). SREBP-1c overexpression induces triglycerides accumulation through increasing lipid synthesis and decreasing lipid oxidation and VLDL assembly in bovine hepatocytes. The Journal of steroid biochemistry and molecular biology.

[34] Brandon EF, Raap CD, Meijerman I, Beijnen JH, Schellens JH (2003). An update on in vitro test methods in human hepatic drug biotransformation research: pros and cons. Toxicology and applied pharmacology.

[35] Dixon JL, Ginsberg HN (1993). Regulation of hepatic secretion of apolipoprotein B-containing lipoproteins: information obtained from cultured liver cells. Journal of lipid research.

